# Evaluating shock index for prediction of adverse maternal outcomes related to postpartum haemorrhage and maternal sepsis in Sierra Leone: a prospective observational cohort study

**DOI:** 10.1016/j.eclinm.2025.103578

**Published:** 2025-10-23

**Authors:** Katy Kuhrt, Foday Janneh, Rossetta Cole, Alexandra Ridout, Cristina Fernandez-Turienzo, Paul T. Seed, Kate Bramham, Andrew H. Shennan

**Affiliations:** a Department of Women and Children's Health, School of Life Course Sciences, Faculty of Life Sciences and Medicine, King's College London, St Thomas' Hospital, Westminster Bridge Road, SE1 7EH, London, UK; b Princess Christian Maternity Hospital, Ministry of Health and Sanitation, Fourah Bay Road, Freetown, Sierra Leone

**Keywords:** Shock index, Postpartum haemorrhage, Maternal sepsis, Vital signs

## Abstract

**Background:**

Postpartum haemorrhage (PPH) and sepsis account for more than half of global maternal deaths. Shock index (SI), the ratio of heart rate to systolic blood pressure, has shown superior prediction of adverse outcomes compared to other vital signs, but most studies are retrospective, with limited evidence in sepsis. We aimed to prospectively evaluate SI for adverse outcome prediction in PPH and sepsis.

**Methods:**

This prospective cohort study was undertaken between March 2022 and July 2023 in three hospitals in Sierra Leone, in 495 and 855 women with PPH or sepsis respectively. ‘First’ and ‘worst’ SI measured in each woman was compared to other vital signs, including modified shock index (MSI), for prediction of outcomes (maternal death; major procedure; lowest haemoglobin <70 g/L; blood transfusion ≥4 international units). Predictive statistics confirmed previously determined SI thresholds (<0.9; 0.9–1.69; ≥1.7), and evaluated SI for rule-out and rule-in of adverse outcomes.

**Findings:**

SI most consistently predicted adverse outcomes compared to other vital signs, including the MSI. In cases of PPH and sepsis, the risk of adverse outcomes increased with higher SI thresholds. For maternal death in PPH, the odds ratios were 5.96 (95% confidence interval [CI]: 1.82–19.53) and 10.02 (95% CI: 3.94–25.50) for the first and worst recorded SI values respectively, and in sepsis, odds ratios were 1.91 (95% CI 0.24–15.26) and 15.4 (95% CI 7.6–31.1) for first and worst recorded SI values, when comparing SI ≥ 1.7 to SI 0.9–1.69. Sensitivity ranged from 88.4% to 95.2%, and negative predictive value ranged from 73.8% to 98.9% across outcomes. The area under the receiver operating characteristic (ROC) curve indicated poorer SI prediction of maternal death in the presence of hypertension (0.86 vs 0.59, p = 0.0020).

**Interpretation:**

SI could effectively rule-in and rule-out adverse outcomes in PPH and sepsis, enabling targeted management, and appropriate resource allocation by health care workers, and informing updates to national definitions and guidelines by policy makers. Future work should further explore the impact of hypertension on SI performance.

**Funding:**

This study was funded by the 10.13039/501100000265UK Medical Research Council (MR/T038942/1) and the 10.13039/501100000272National Institute for Health and Care Research (NIHR133232).

**Research in context**

**Evidence before this study**

We searched PubMed for original articles published in English between September 30, 2019 to September 30, 2024, with the search terms ‘postpartum haemorrhage OR maternal sepsis OR puerperal sepsis AND shock index’. We identified 20 studies (17 of which were undertaken in high-income settings), which examine shock index prediction in postpartum haemorrhage or maternal sepsis. The majority of studies are retrospective and evaluate shock index for prediction of postpartum haemorrhage diagnosis or blood loss volume rather than adverse clinical outcomes. Prior to this study, the largest prospective study validated rule-in and rule-out shock index thresholds for prediction of adverse clinical outcomes in postpartum haemorrhage and sepsis, and found that shock index was the most consistently reliable predictor compared to other vital signs but it had small numbers of women in the highest risk shock index category.

**Added value of this study**

To our knowledge, this is the largest prospective study evaluating SI for prediction of adverse clinical outcomes in PPH and maternal sepsis, and to include an assessment of modified shock index. In three maternity settings in Sierra Leone, we showed that shock index is the most consistent predictor of adverse clinical outcomes in a high-risk cohort of women with postpartum haemorrhage or sepsis. For the first time we have prospectively demonstrated in an obstetric cohort with bleeding or sepsis that modified shock index did not improve prediction of adverse outcomes. Very few studies of a similar size have been carried out in maternity populations in a low and middle-income settings, where the potential for impact is greatest.

**Implications of all the available evidence**

We have shown that shock index, a simple concept, that does not require expensive, complicated equipment, could predict important clinical outcomes, including maternal death, related to postpartum haemorrhage and maternal sepsis. With further evaluation, this could enable targeted allocation of scarce resources, especially valuable in high-burden settings, like Sierra Leone, where stock-outs may limit availability of life-saving interventions.

## Introduction

Bleeding and infection account for more than half of maternal deaths globally,^1^ the majority of which (>99%) occur in low-and middle-income settings (LMICs),^2^ and for every woman who dies at least 10 are affected by morbidity, including peripartum hysterectomy or shock-related organ system injuries.^3^ Most deaths are associated with delayed or missed diagnoses and subsequent timely intervention, partly due to a lack of consistent definitions for postpartum haemorrhage (PPH) and sepsis. Early identification of haemodynamic compromise based on vital signs measurement is critical in preventing maternal mortality and morbidity.

Shock index (SI) is the ratio of heart rate (HR) to systolic blood pressure (SBP) and has been shown to be an early predictor of the need for hospital admission, massive blood transfusion, major trauma protocol activation, intensive care unit (ICU) admission and death in non-obstetric patients attending emergency triage,^4^^,^^5^ trauma,^6^^,^^7^ and sepsis,^8^ including in COVID-19 patients.^9^ According to retrospective cohorts of women in high and LMICs, SI has been shown to be the most consistent predictor of outcome compared to individual conventional vital signs, with thresholds (<0.9; 0.9–1.69 and ≥ 1.7) indicating increased risk of adverse outcomes.^10^^,^^11^ A prospective LMIC analysis validated SI thresholds of women with PPH and sepsis and showed that SI < 0.9 performed well as a rule out test and 0.9–1.69 and ≥ 1.7 indicated increased risk of adverse outcomes, although larger prospective studies are needed to substantiate these findings, particularly with respect to maternal sepsis where the number of women in the higher threshold categories was small.^12^ More recently, retrospective studies have shown improved prediction of adverse outcomes with ‘modified shock index’ (MSI) (HR/mean arterial pressure) in non-obstetric ICU, emergency,^13^ cardiac^14^ and septic^15^ patients, but this has not prospectively determined, including in obstetric patients.

To date, the evidence suggests that SI could help detection and management of haemodynamic compromise in PPH and sepsis, but the potential clinical utility of SI needs further exploration, including in larger septic cohorts, and in women with common co-morbid conditions, e.g.: hypertensive disorders of pregnancy (HDP), the second leading cause of global maternal mortality, where alternative thresholds or management pathways may be appropriate,^16^ to better understand how it can be pragmatically integrated into clinical care pathways.

We will undertake a large, prospective, multi-site evaluation of SI for prediction of adverse clinical outcomes in PPH and maternal sepsis, including maternal death; major surgical or invasive procedure; lowest haemoglobin following diagnosis <70 g/L; blood transfusion ≥4 international units (IU) requested; and blood transfusion ≥4 IU given, in maternity healthcare settings in Sierra Leone.

## Methods

This prospective cohort study was undertaken between March 2022 and July 2023 at three maternity units in Sierra Leone: Princess Christian Maternity Hospital (tertiary referral hospital, Freetown), Kabala and Moyamba Government hospitals (district hospitals). Consecutive women with either or both PPH or maternal sepsis (antepartum or postpartum) during their admission up until discharge from hospital were eligible. PPH was defined as an estimated blood loss (EBL) ≥500 mL following vaginal delivery and ≥1000 mL following caesarean delivery, or based on documentation of PPH in patients' medical notes. This pragmatic approach was taken given unreliable documentation of EBL. Diagnosis of maternal sepsis diagnosis was based on temperature ≥37.5 and or clinical features determined by women's healthcare providers and documented in her notes.

Vital signs monitoring was routinely performed using the Microlife CRADLE Vital Signs Alert (VSA), a vital signs device that measures BP and HR, and calculates SI, is suitable for use in LMICs, and is validated as accurate for use in pregnancy, including in Pre-eclampsia and hypotension.^17^ The device incorporates a traffic light early-warning system triggering a green, yellow or red light according to hypertension or previously determined SI thresholds (<0.9; 0.9–1.69 and ≥ 1.7).^11^ Clinicians used the HR and BP measurements to determine clinical decisions, and managed women according to local practice. They were not masked to the traffic light alerts but were not trained specifically to respond to them.

The ‘first’ and ‘worst’ sets of BP and HR were collected from the medical notes. The ‘first’ set was defined as those documented at the time of initial clinical assessment and diagnosis and the ‘worst’ as the set corresponding to the highest SI, documented at any time between initial clinical assessment and discharge (or maternal death). The predefined outcomes were maternal death; major surgical or invasive procedure; lowest haemoglobin following diagnosis <70 g/L; blood transfusion ≥4 IU requested; and blood transfusion ≥4 IU given (both were collected due to anticipated challenges in availability of blood). We did not include High Dependency Unit as an outcome because two out of three study sites did not have HDU facilities. Major surgical or invasive procedures were defined as: perineal/cervical tear repair; manual removal of placenta (MROP); uterine balloon tamponade; evacuation of the uterus (EOU); emergency laparotomy for haemorrhage; exploratory laparotomy for ectopic pregnancy; emergency hysterectomy; or other procedures related to sepsis). We have previously demonstrated that SI is not influenced by regional analgesia so this data was not collected.^18^ Data were extracted from patient notes by local and external researchers, and data quality checks carried out by an external researcher. Discrepancies were adjudicated by an obstetrician. Women with missing vital signs were excluded: women with missing outcomes were included but not analysed for the outcome for which the data was missing.

### Ethics

The study was approved by Sierra Leone Ethics and Scientific Review Committee (009/12/2022) and King's College London Research Ethics Committee (LRS-19/20-18/45). Institutional level agreement for the study was given at all three sites, Princess Christian Maternity Hospital, Moyamba Government Hospital and Kabala Government Hospital and individual level consent was not required because vital signs assessment using Microlife CRADLE VSA was already part of routine care and no identifiable data was collected.

### Statistical analyses

Predefined analysis aimed to determine whether SI was a consistent predictor of adverse outcomes. This was calculated using area under the receiver operator curve (AUROC). We used 95% confidence intervals to assess the ability of the ‘first’ and ‘worst’ SI, MSI and conventional vital signs (HR, systolic BP, diastolic BP, mean arterial pressure, and pulse pressure) for predicting the predefined outcomes. Mean arterial pressure was defined as (2× diastolic BP + systolic BP)/3 and pulse pressure was defined as systolic BP—diastolic BP. Predictor equality of AUROCs across the outcomes was tested using unadjusted chi-square analysis.

Our predefined analysis was to calculate sensitivities, specificities and positive and negative predictive values (NPV) for four potential SI thresholds (0.7, 0.9, 1.4, 1.7), identified from the literature,^11^^,^^12^^,^^19^^,^^20^ to confirm optimal SI categories to rule-out and rule-in adverse outcomes.

The ability of the ‘first’ and ‘worst’ SI categories (SI < 0.9, SI 0.9–1.69 and SI ≥ 1.7) to predict the risk of each predetermined outcome was evaluated using post-test probabilities for each category, odds ratios of SI 0.9–1.69 vs SI < 0.9 and SI ≥ 1.7 vs SI 0.9–1.69, and non-parametric trend testing of change in risk across the SI categories. To calculate the post-test probabilities of each event for SI each category, the total number of events in each category was divided by the total number of women in that SI category, with 95% confidence intervals calculated using the exact Clopper-Pearson method.^21^ Post-test probability was used to evaluate the performance of the three categories (SI < 0.9, SI 0.9–1.69, and SI ≥ 1.7), rather than traditional predictive testing using sensitivity, specificity, positive predictive value (PPV) and NPV, which is only appropriate when testing one threshold or two categories. Sensitivity, specificity, PPV, and NPV were used to evaluate the test performance of thresholds <0.9 as a rule-out test and ≥1.7 as a rule-in test at ‘worst’ vital signs measurement following diagnosis for prediction of adverse clinical outcomes. Shock index performance for prediction of maternal death in women with and without hypertensive status was evaluated. The exact shock index was used to calculate AUROC with 95% CIs which were then compared using chi-squared analysis. Separate analysis was performed for bleeding and maternal sepsis, as crossover was anticipated to be relatively small. Women with both diagnoses were included in both groups.

A post-hoc power calculation was performed for maternal outcome. Equal numbers of women had first SI above and below 0.9. The overall rate of maternal deaths was 4%. To detect a difference of 5% in maternal death rates (1.5% vs 6.5%), for SI < 0.9 vs SI ≥ 0.9 we needed 480 women for 80% power, and 642 for 90% power. Complete data was available for 495 women in the PPH group (power = 81.1%), and 855 in the sepsis group (power = 96.2%). Statistical analysis was performed in the Statistical package STATA, version 18 (StataCorp, College Station, Texas, UK). The study was reported in accordance with both Strengthening the Reporting of Observational studies in Epidemiology (STROBE^22^) and Standards for Reporting Diagnostic Accuracy (STARD)^23^ guidelines.

### Role of the funding source

The funders of the study had no role in study design, data collection, data analysis, data interpretation, or writing of the report. The corresponding author had full access to all the data in the study and had final responsibility for the decision to submit for publication.

## Results

**Fig. 1:**
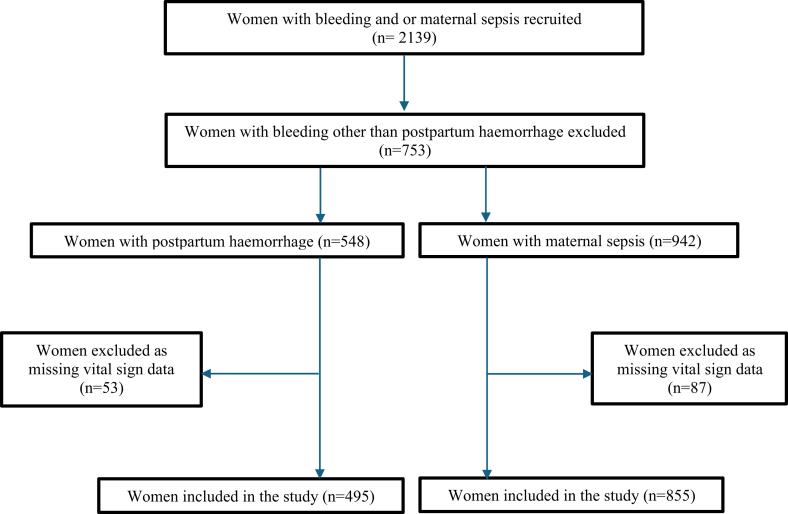
Flow diagram of participants.

A total of 495 women with PPH and 855 women with maternal sepsis were included in the planned analyses (Fig. 1). 104 women had both diagnoses and were included in both groups. Participant characteristics are shown in Table 1. Median estimated blood loss was lower than expected in the PPH group: 500 mL (300–800 mL), and inconsistent with high rates of transfusion indicating severe haemorrhage, which may reflect underestimation of blood loss.

**Table 1:** Demographic, admission, delivery, perinatal outcomes and haemorrhage/sepsis details for postpartum haemorrhage and maternal sepsis.

	Postpartum haemorrhage (n = 495)	Maternal sepsis (n = 855)
**Demographic details**		
Age, years	25.8 ± 6.3	25.3 ± 5.8
Multiparous, n	366/477, 76.7%	519/820, 63.3%
**Delivery details**		
Gestation at delivery, weeks	36.3 ± 4.1	35.2 ± 4.8
Preterm birth <34 weeks, n	33/232, 14%	79/358, 22.1%
Preterm birth <37 weeks, n	123/232, 53.0%	212/358, 59.2%
Mode of delivery: Caesarean section, n	89/259, 34.4%	217/377, 57.6%
**Diagnosis details**		
Gestation at diagnosis, weeks	35.9 ± 4.8	30.4 ± 9.1
Diagnosis made antenatally	N/A	199/355, 56.1%
Estimated blood loss, mL	500 (300–800)	400 (200–600)
Intravenous antibiotics given, n	329/492, 66.9%	647/847, 76.4%
**Perinatal outcomes (where recorded)**		
Stillbirth, n	58/238, 23.1%	88/379, 23.2%
Neonatal death before discharge, n	9/214, 4.2%	16/324, 4.9%

Mean ± standard deviation, median (interquartile range) and n, percentage are shown. NA, not applicable.

Vital sign results are shown in Table S1. Three hundred and sixty-six women with PPH (73.9%) had a ‘worst’ SI following diagnosis 0.9–1.69, and 42 women (8.5%) had a ‘worst’ SI following diagnosis *≥*1.7. Similar proportions of women fell into each SI category in the maternal sepsis group.

Table 2 shows the incidence of each outcome. Twenty-one women (4.2%) with PPH and 43 women (5.2%) with maternal sepsis died whilst admitted. Forty-three percent and 34.6% of women had a lowest haemoglobin less than 70 g/dL in the PPH and sepsis groups respectively, with a similar drop in haemoglobin (5.2 vs 5.11 g/dL). Despite this, half as many women in the sepsis group received a blood transfusion (30.4% vs 63.6%). Twenty-six women in the PPH group and 28 women in the maternal sepsis group had blood requested but not given, which highlights the challenges related to availability of blood transfusion in this setting. Only two women (0.4%) in the PPH group and 1 woman (0.1%) in the maternal sepsis group underwent emergency hysterectomy, likely reflective of skills gaps and human resource shortages rather than lack of clinical indication.

**Table 2:** Mean ± SD or n, % of adverse outcomes for the postpartum haemorrhage and maternal sepsis groups.

Outcomes	Postpartum haemorrhage (n = 495)	Maternal sepsis (n = 855)
Maternal death	21/495, 4.2%	43/855, 5.2%
Lowest haemoglobin following diagnosis, g/L	72.4 ± 30.07	80.3 ± 32.8
Lowest haemoglobin <70 g/L following diagnosis	179/410, 437%	195/563, 34.6%
Drop in haemoglobin, g/L	5.2 ± 13.8	5.11 ± 13.8
Drop in haemoglobin ≥20 g/L	93/405, 23.0%	116/555, 20.9%
Blood transfusion requested	340/495, 68.7%	288/855, 33.7%
Number of IU requested	2.7 ± 1.08	2.7 ± 1.2
Blood transfusion given	315/495, 63.6%	260/855, 30.4%
Number of IU given	2.7 ± 1.09	2.8 ± 1.2
Blood transfusion ≥4 units requested, n (% of all women)	86/495, 17.4%	82/855, 9.6%
Blood transfusion ≥4 units given, n (% of all women)	82/495, 16.6%	80/855, 9.4%
Any Major surgical or invasive procedure (composite)	198/495, 40.0%	113/855, 13.2%
Individual components of composite:		
Perineal/cervical tear repair	78/495, 15.8%	30/855, 3.5%
Manual removal of placenta	43/495, 8.7%	17/855, 2.0%
Uterine balloon tamponade	4/495, 0.8%	1/855, 0.1%
Evacuation of the uterus	93/495, 18.8%	40/855, 4.7%
Exploratory lap for bleeding	6/495, 1.2%	9/855, 1.1%
Emergency laparotomy for uterine rupture	6/495, 1.2%	9/855, 1.1%
Emergency lap for ectopic	N/A	6/855, 0.7%
Emergency hysterectomy	2/495, 0.4%	1/855, 0.1%
Other procedures related to sepsis	2/495, 0.4%	18/855, 2.1%

Mean ± standard deviation, median (interquartile range) and n, percentage are shown. NA, not applicable.

According to area under the receiver operating characteristic curve (AUROC) values, SI at both ‘first’ and ‘worst’ vital sign measurements following diagnosis was amongst the top two best performing vital signs for every adverse outcome for both PPH and maternal sepsis.

For predicting maternal death, SI had an AUROC of 0.80 (95% CI 0.69–0.91) in women with PPH and 0.76 (95% CI 0.67–0.73) for women with maternal sepsis (Table 3).

**Table 3:** Area under the receiver operating characteristic curve (AUROC) for ‘worst’ vital sign parameters to predict adverse clinical outcomes among women with postpartum haemorrhage and maternal sepsis.

	SI	MSI	HR	SBP	DBP	MAP	PP
**Maternal death**							
PPH	**0.80 (0.69–0.91)**	**0.78 (0.66–0.90)**	0.70 (0.56–0.84)	0.74 (0.61–0.87)	0.70 (0.58–0.83)^a^	0.73 (0.61–0.86)	0.69 (0.56–0.81)
Maternal sepsis	**0.76 (0.67–0.85)**	0.74 (0.65–0.84)	**0.81 (0.74–0.88)**	0.63 (0.52–0.74)^a^	0.59 (0.48–0.69)^a^	0.61 (0.50–0.71)^a^	0.64 (0.55–0.73)^a^
**Major surgical or invasive procedure**							
PPH	**0.59 (0.54–0.64)**	**0.59 (0.54–0.64)**	0.56 (0.51–0.61)	0.56 (0.51–0.62)	0.56 (0.51–0.61)	0.57 (0.51–0.62)	0.52 (0.47–0.58)^a^
Maternal sepsis	**0.64 (0.59–0.69)**	0.63 (0.58–0.68)	**0.63 (0.58–0.69)**	0.55 (0.49–0.61)^a^	0.52 (0.47–0.58)^a^	0.53 (0.48–0.59)^a^	0.58 (0.52–0.64)
**Lowest HB <70 g/L**							
PPH	**0.66 (0.61–0.72)**	**0.66 (0.60–0.71)**	0.58 (0.53–0.64)^a^	0.64 (0.59–0.69)	0.62 (0.57–0.68)	0.63 (0.58–0.69)	0.58 (0.52–0.63)^a^
Maternal sepsis	**0.61 (0.56–0.66)**	**0.61 (0.56–0.66)**	0.58 (0.53–0.63)	0.56 (0.51–0.61)^a^	0.56 (0.51–0.61)^a^	0.56 (0.51–0.61)^a^	0.52 (0.47–0.57)^a^
**Blood transfusion requested ≥ 4 IU**							
PPH	0.65 (0.59–0.72)	0.64 (0.57–0.70)	0.62 (0.56–0.69)	0.59 (0.53–0.66)^a^	0.59 (0.52–0.66)^a^	0.59 (0.52–0.66)^a^	0.55 (0.48–0.62)^a^
Maternal sepsis	**0.65 (0.59–0.72)**	0.64 (0.57–0.71)	**0.65 (0.59–0.71)**	0.57 (0.51–0.64)^a^	0.57 (0.50–0.64)^a^	0.57 (0.51–0.64)^a^	0.54 (0.48–0.61)^a^
**Blood transfusion given ≥4 IU**							
PPH	**0.63 (0.57–0.69)**	**0.62 (0.55–0.68)**	0.60 (0.54–0.67)	0.58 (0.51–0.64)^a^	0.57 (0.50–0.64)	0.57 (0.50–0.64)^a^	0.54 (0.46–0.61)^a^
Maternal sepsis	**0.65 (0.59–0.72)**	0.64 (0.57–0.71)	**0.65 (0.59–0.71)**	0.57 (0.50–0.64)^a^	0.57 (0.50–0.64)^a^	0.57 (0.51–0.64)^a^	0.54 (0.47–0.61)^a^

AUROC values given as AUROC (95% CI). In bold: highest two AUROC values for each outcome. Results of significance testing for equality of AUROCs using unadjusted chi-square test, with shock index as a reference.

MSI, Modified Shock Index; MAP, Mean arterial pressure; PP, Pulse pressure.

a Significantly worse than shock index (P < 0.05).

Shock index threshold of 0.9 maintained reasonable sensitivity (90.0%) with clinical practicality with a superior NPV (98.3%) and PPV (4.2%) compared to 0.7 (97.6% and 3.7% respectively). For ‘rule-in’ 1.4 balanced specificity (84.1%) and NPV (98.3%), and 1.7 further improved specificity (96.0%) with minimal impact on NPV (97.7%). Therefore we were able to confirm our previously determined thresholds of 0.9 and 1.7 as optimal for ‘rule-out’ and ‘rule in’ of adverse outcomes respectively.

Tables 4 and 5 show the frequency and percentage of outcomes across SI categories, and post-test probabilities, odds ratios and the non-parametric trend test for both groups. In PPH, statistical testing for trend showed a significant increase in risk with higher SI categories at ‘first’ SI measurement for: maternal death, lowest HB less than 70 g/dL, at least 4 IU blood transfusion requested and received, and a non-significant but upward trend for major surgical or invasive procedure. For the ‘worst’ SI, all outcomes showed a significant increase in risk except at least 4 IU blood transfusion given, which showed a non-significant upward trend. For the sepsis group, statistical test for trend showed a significant increase in risk with increasing ‘first’ SI categories for lowest HB less than 70 g/dL, at least 4 IU blood transfusion requested and given, and a non-significant increase in risk for the remaining outcomes, and for ‘worst’ SI, there was a significant increase in risk across all outcomes except lowest HB less than 70 g/dL.

**Table 4:** Frequency and post-test probability of outcomes in women with “first” and “worst” SI < 0.9, SI 0.9–1.69, and SI ≥ 1.7 following diagnosis of postpartum haemorrhage, odds ratios of SI 0.9–1.69 vs SI < 0.9 and SI ≥ 1.7 vs SI 0.9–1.69 and non-parametric trend test for worsening SI category (SI < 0.9 to SI 0.9–1.69 to SI ≥ 1.7).

	Maternal death	Major surgical or invasive procedure	Lowest haemoglobin <70 g/L	Blood transfusion requested *≥*4 IU	Blood transfusion given *≥*4 IU
**First vital signs**					
Post-test probability					
SI < 0.9 (236)	5, 2.12 (0.69–4.87)	92, 38.98 (32.7–45.5)	60, 32.79 (26.0–40.0)	30, 12.7 (8.7–17.6)	30, 12.7 (8.7–17.6)
SI 0.9–1.69 (237)	12, 5.06 (2.64–8.67)	95, 40.08 (33.8–46.6)	104, 50.24 (43.2–57.2)	48, 20.25 (15.3–25.9)	46, 19.4 (14.57–25.02)
SI ≥ 1.7 (22)	4, 18.18 (5.18–40.2)	11, 50.00 (28.2–71.8)	15, 75.00 (50.8–91.3)	8, 36.36 (17.1–59.3)	6, 27.27 (10.7–50.2)
SI 0.9–1.69 vs SI < 0.9 OR	**3.04** (1.10–8.44)	1.08 (0.76–1.55)	**2.26** (1.51–3.38)	**1.89** (1.16–3.1)	**1.72** (1.1–2.81)
SI ≥ 1.7 vs SI 0.9–1.69 OR	**5.96** (1.82–19.53)	1.53 (0.65–3.60)	**4.13** (1.47–11.6)	**2.39** (1.17–7.13)	1.96 (0.74–5.17)
P-value	**0.0018**	0.4654	**0.0001**	**0.0018**	**0.0167**
**Worst vital signs**					
Post-test probability					
SI < 0.9 (87)	1, 1.15 (0.02–6.23)	24, 27.59 (18.54–38.21)	16, 23.19 (13.93–34.9)	10, 11.49 (5.65–20.12)	10, 11.49 (5.65–20.12)
SI 0.9–1.69 (366)	11, 3.01 (1.51–5.34)	151, 41.26 (36.1–46.4)	140, 46.20 (40.5–51.9)	63, 17.21 (13.49–21.48)	62, 16.94 (13.2–21.2)
SI ≥ 1.7 (42)	9, 21.4 (10.30–36.81)	23, 54.76 (38.7–70.2)	23, 60.53 (43.38–75.96)	13, 30.95 (17.62–47.09)	10, 23.81 (12.05–39.45)
SI 0.9–1.69 vs SI < 0.9 OR	4.43 (0.59–33.48)	**1.95** (1.17–3.25)	**3.03** (1.67–5.52)	1.76 (0.87–3.57)	1.65 (0.81–3.34)
SI ≥ 1.7 vs SI 0.9–1.69 OR	**10.02** (3.94–25.50)	**1.92** (1.02–3.63)	**2.12** (1.07–4.20)	**2.33** (1.16–4.70)	1.65 (0.79–3.51)
P-value	**0.0001**	**0.0019**	**0.0001**	**0.0106**	0.0728

Post-test probability values are given as n, % (95% confidence interval), odds ratios given as OR (95% confidence intervals), values in bold indicate statistical significance. All P-values are based on the non-parametric test for trend.

**Table 5:** Frequency and post-test probability of outcomes in women with “first” and “worst” SI < 0.9, SI 0.9–1.69, and SI ≥ 1.7 following diagnosis of sepsis, odds ratios of SI 0.9–1.69 vs SI < 0.9 and SI ≥ 1.7 vs SI 0.9–1.69 and non-parametric trend test for worsening SI category (SI < 0.9 to SI 0.9–1.69 to SI ≥ 1.7).

	Maternal death	Major surgical procedure (any)	Lowest haemoglobin <70 g/dL	Blood transfusion requested *≥*4 IU	Blood transfusion given *≥*4 IU
**First vital signs**					
Post-test probability					
SI < 0.9 (401)	19, 4.74 (2.88–7.30)	47, 11.72 (8.74–15.3)	68, 29.1 (23.3–35.3)	30, 7.48 (5.1–10.5)	29, 7.23 (4.89–10.2)
SI 0.9–1.69 (443)	23, 5.19 (3.32–7.69)	63, 14.22 (11.1–17.8)	125, 39.06 (33.7–44.6)	50, 11.29 (8.49–14.6)	49, 11.06 (8.29–14.4)
SI ≥ 1.7 (11)	1, 9.09 (0.22–41)	3, 27.27 (6.02–60.9)	2, 22.22 (2.8–60.0)	2, 18.18 (2.2–51.8)	2, 18.18 (2.28–51.8)
*SI 0.9–1.69 vs SI < 0.9 OR*	1.12 (0.61–2.10)	1.28 (0.86–1.91)	**1**.**53 (1**.**07–2**.**20)**	1.60 (0.99–2.56)	**1**.**62 (1**.**01**–**2**.**62)**
*SI ≥ 1*.*7 vs SI 0*.*9–1*.*69 OR*	1.91 (0.24–15.26)	2.50 (0.65–9.57)	0.53 (0.11–2.59)	2.12 (0.45–9.99)	2.18 (0.46–10.28)
P-value	0.6293	0.1454	**0**.**0427**	**0**.**0369**	**0**.**0332**
**Worst vital signs**					
Post-test probability					
SI < 0.9 (165)	4, 2.42 (0.66–6.1)	11, 6.67 (3.4–11.6)	22, 26.19 (17.2–36.9)	8, 4.85 (2.1–9.3)	8, 4.85 (2.1–9.3)
SI 0.9–1.69 (640)	22, 3.44 (2.16–5.20)	90, 14.06 (11.4–16.99)	156, 36.03 (31.5–40.7)	62, 9.69 (7.50–12.2)	61, 9.53 (7.3–12.1)
SI ≥ 1.7 (50)	17, 34 (21.2–48.8)	12, 24 (13.1–38.1)	17, 36.96 (23.2–52.5)	12, 24 (13.0–38.10)	11, 22 (11.52–35.96)
*SI 0.9–1.69 vs SI < 0.9 OR*	2.4 (0.85–6.84)	**2.43** (1.27–4.64)	1.59 (0.95–2.68)	**2**.**35** (1.11–4.99)	**2**.**29** (1.08–4.85)
*SI ≥ 1*.*7 vs SI 0*.*9–1*.*69 OR*	**15**.**4** (7.6–31.2)	**2**.**20** (1.11–4.35)	1.12 (0.59–2.09)	**3**.**31** (1.66–6.64)	**3**.**01** (1.47–6.14)
P-value	**0**.**0001**	**0**.**0007**	0.1286	**0**.**0003**	**0**.**0008**

Post-test probability values are given as n, % (95% confidence interval), odds ratios given as OR (95% confidence intervals), values in bold indicate statistical significance. All P-values are based on the non-parametric test for trend.

Both ‘first’ and ‘worst’ SI ≥ 1.7 were associated with high risks of adverse outcomes in both groups. For example, in PPH, of the 22 women with ‘first’ SI ≥ 1.7, nearly a quarter (4 of 22) died, half (11 of 22) underwent a major surgical or invasive procedure, and a third (8 of 22) required at least four IU blood transfusion. Similar proportions of women with PPH experienced adverse outcomes if their ‘worst’ SI was ≥1.7. In the maternal sepsis group, SI was ≥1.7 was also associated with very high risks, for ‘first’ and ‘worst’ SI. In the group of women with ‘worst’ SI ≥ 1.7, one third (17 of 50) died.

Tables 6 and 7 show the sensitivity, specificity, PPV and NPVs for ‘worst’ SI less than 0.9 and ≥1.7 respectively. For ‘worst’ SI < 0.9 sensitivity was consistently high, e.g.: 95.2% (95% CI 76.2–99.9) for maternal death, where 20 of 21 women who died were correctly identified (Table 6), whilst Table 7 shows specificities of at least 92% for every outcome across both groups indicating a very high risk of an adverse event with a SI ≥ 1.7.

**Table 6:** Test performance statistics for “worst” SI < 0.9 in prediction of adverse outcomes in women with postpartum haemorrhage or maternal sepsis.

Outcomes	PPH	Sepsis	PPH	Sepsis	PPH	Sepsis	PPH	Sepsis	PPH	Sepsis
	Maternal death		Major surgical or invasive procedure		Lowest haemoglobin <70 g/dL		Blood transfusion requested *≥*4 IU		Blood transfusion given *≥*4 IU	
	n = 21	n = 43	n = 198	n = 113	n = 179	n = 195	n = 86	n = 82	n = 82	n = 80
Sensitivity (%) n/N	95.2 (76.2–99.9) 20/21	90.7 (77.9–97.4) 39/43	87.9 (82.5–92.1) 174/198	90.3 (83.2–95.0) 102/113	91.1 (85.9–94.8) 163/179	88.8 (83.4–92.8) 173/195	88.4 (79.7–94.3) 76/86	90.2 (81.7–95.7) 74/82	87.8 (78.7–94.0) 72/82	90.0 (81.2–95.6) 72/80
Specificity (%) n/N	18.1 (14.8–21.9) 86/474	19.8 (17.1–22.7) 161/812	21.2 (16.7–26.3) 63/297	20.8 (17.9–23.9) 154/742	22.9 (17.7–28.9) 53/231	16.8 (13.2–21.1) 62/368	18.8 (15.2–23.0) 77/409	20.3 (17.5–23.3) 157/773	18.6 (15.0–22.7) 77/413	20.3 (17.5–23.3) 157/775
PPV (%) n/N	4.9 (3.0–7.5) 20/408	5.7 (4.0–7.6) 39/690	42.6 (37.8–47.6) 174/408	14.8 (12.2–17.7) 102/690	47.8 (42.4–53.2) 163/179	36.1 (31.8–40.6) 173/479	18.6 (15.0–22.8) 76/408	10.7 (8.5–13.3) 74/690	17.6 (14.1–21.7) 72/408	10.4 (8.3–13.0) 72/690
NPV (%) n/N	98.9 (93.8–100) 86/87	97.6 (93.9–99.3) 161/165	72.4 (61.8–81.5) 63/87	93.3 (88.4–96.6) 154/165	76.8 (65.1–86.1) 53/69	73.8 (63.1–82.8) 62/84	88.5 (79.9–94.3) 77/87	95.2 (90.7–97.9) 157/165	88.5 (79.9–94.3) 77/87	95.2 (90.7–97.9) 157/165

PPH, postpartum haemorrhage; PPV, positive predictive value; NPV, negative predictive value.

**Table 7:** Test performance statistics for “worst” SI ≥ 1.7 in prediction of adverse outcomes in women with postpartum haemorrhage or maternal sepsis.

Outcomes	PPH	Sepsis	PPH	Sepsis	PPH	Sepsis	PPH	Sepsis	PPH	Sepsis
	Maternal death		Major surgical or invasive procedure		Lowest haemoglobin <70 g/dL		Blood transfusion requested *≥*4 IU		Blood transfusion given *≥*4 IU	
	n = 21	n = 43	n = 198	n = 113	n = 179	n = 195	n = 86	n = 82	n = 82	n = 80
Sensitivity (%) n/N	42.9 (21.8–66) 9/21	39.5 (25–55.6) 17/43	11.6 (7.1–6.9) 23/198	10.6 (5.6–17.8) 12/113	12.8 (8.3–18.7) 23/179	8.7 (5.2–13.6) 17/195	15.1 (8.3–24.5) 13/86	14.6 (7.8–24.2) 12/82	12.2 (6.0–21.3) 10/82	13.8 (7.1–23.3) 11/80
Specificity (%) n/N	93.0 (90.4–95.2) 441/474	95.9 (94.3–97.2) 779/812	93.6 (90.2–96.1) 278/297	94.9 (93–96.4) 704/742	93.5 (89.5–96.3) 216/231	92.1 (88.9–94.7) 339/368	92.9 (90.0–95.2) 380/409	95.1 (93.3–96.5) 735/773	92.3 (89.2–94.6) 381/413	95.0 (93.2–96.4) 736/775
PPV (%) n/N	21.4 (10.3–36.8) 9/21	34.0 (21.2–48.8) 17/50	54.8 (38.7–70.2) 23/42	24.0 (13.1–38.2) 12/50	60.5 (43.4–76.0) 23/38	37.0 (23.2–52.5) 17/46	31.0 (17.6–47.1) 13/42	24.0 (13.1–38.2) 12/50	23.8 (12.1–39.5) 10/42	22.0 (11.5–36.0) 11/50
NPV (%) n/N	97.4 (95.4–98.6) 441/474	96.8 (95.3–97.9) 779/805	61.4 (56.7–65.9) 278/453	87.5 (85–89.7) 704/805	58.1 (52.9–63.1) 216/372	65.6 (61.3–69.7) 339/517	83.9 (80.2–87.2) 77/87	91.3 (89.1–93.2) 735/805	84.1 (80.4–87.4) 381/453	91.4 (89.3–93.3) 736/805

PPH, postpartum haemorrhage; PPV, positive predictive value; NPV, negative predictive value.

**Fig. 2:**
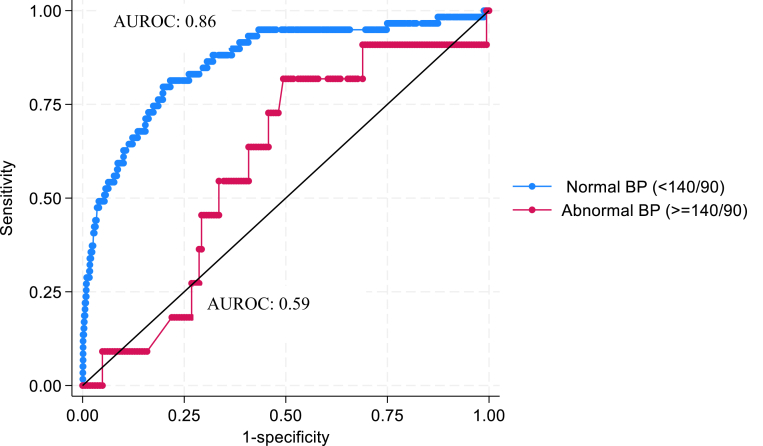
Receiver operating characteristic curve for ‘worst’ shock index predicting maternal death in women with hypertension in addition to a diagnosis of postpartum haemorrhage or sepsis and women without. AUROC, area under the receiver operating characteristic curve; BP, blood pressure.

The AUROC for prediction of maternal death in women who were hypertensive in addition to their diagnosis of either sepsis and/or PPH was significantly worse compared to women who were not hypertensive (AUROC 0.59 vs 0.86, p = 0.0020) (Fig. 2).

## Discussion

SI was the most consistent predictor, compared with other vital signs, and MSI, of all adverse clinical outcomes in PPH and maternal sepsis, the two leading causes of maternal mortality globally. Previously determined ‘abnormal’ SI thresholds (0.9–1.69; ≥1.7) were confirmed, and were significantly associated with adverse clinical outcomes in PPH and sepsis groups, in this larger prospectively collected data set in a high risk cohort, including 64 maternal deaths. The SI category at ‘first’ vital signs measurement was predictive of adverse outcomes, including maternal death, lowest HB < 70 g/L and blood transfusion, following PPH diagnosis, with a stepwise increase in risk from SI < 0.9 to SI 0.9–1.69, to SI ≥ 1.7. The SI category at ‘worst’ vital signs measurement was predictive of all adverse clinical outcomes for both groups. Our results indicate that SI prediction may be inferior in hypertensive women with PPH or sepsis. To our knowledge, this is the largest prospective study evaluating SI for prediction of adverse clinical outcomes in PPH and maternal sepsis. This evidence is timely, and well-aligned with the recently published WHO PPH Roadmap, which highlighted SI as one of 15 priority research topics to be addressed in order to expedite progress towards reducing mortality from PPH. To improve generalisability of the results, multiple centres (tertiary urban and large secondary district) hospital settings, limited exclusion criteria, and multiple clinically important severe outcomes were used to assess prediction.

Some outcomes were resource dependent, which is why we included need for blood transfusion (‘blood transfusion ≥4 IU requested’), as well as receiving it (‘blood transfusion ≥4 IU given’). Furthermore, withholding interventions due to lack of availability would weaken test performance of SI, and in spite of this we found significant associations between SI and adverse outcomes, such that results remain valid. Arguably, associations may have been even stronger if resource-dependent ‘process measures’ had not been used as outcomes, as seen for maternal death (e.g.: AUROC in PPH, 0.8), or in better resourced settings. We were unable to reliably determine timing of clinical events to vital sign measurement. The study site is the only tertiary maternity hospital in Sierra Leone, meaning that many study participants were transferred from home or a community health facility, often with limited information about the exact timing of prior events. Added to this, clinical documentation at the study hospital was often missing timings, such that we were not able to include more precise estimates than average times from diagnosis to first and worst shock index presented in Table S1. However, despite these limitations, which applied to all vital signs and measurements collected, SI remained the most consistent predictor of outcome, as demonstrated in Table 3, and therefore this does not prevent us from concluding that SI likely has clinical utility in this setting. We acknowledge the importance of determining more granular detail in relation to vital sign measurement and clinical outcome, and this is planned in a study due to start imminently.

Our research team previously evaluated SI retrospectively, then prospectively, in PPH and sepsis in LMICs, and identified SI thresholds for ‘rule-in’ and ‘rule-out’ of adverse events.^11^ They showed that ‘first’ SI following diagnosis was most useful, whilst in sepsis, the ‘worst’ SI measurement was more predictive, although their findings were limited by the small numbers (n = 6) in the ‘worst’ SI category (SI ≥ 1.7).^12^ Our findings, in a larger cohort of women, confirmed this observation, which likely reflects the more rapid deterioration seen in haemorrhage compared to sepsis. In a retrospective secondary analysis of the TRAAP study, a randomized, double-blind, placebo-controlled trial that investigated the effectiveness of tranexamic acid (TXA) in preventing PPH after vaginal delivery,^24^ Madaar et al. concluded that SI did not accurately predict PPH diagnosis, but they were limited in their ability to predict clinically important endpoints, given small numbers of adverse outcomes in their cohort.^25^ Our study is unique in its ability to demonstrate robust prediction for death and other severe outcomes given the tragically high mortality and morbidity burden in this setting.

More recently, MSI, the ratio between HR and mean arterial blood pressure, has been found to be superior to SI for prediction of adverse clinical outcomes in non-obstetric emergency department,^13^ cardiac^14^ and septic^9^^,^^15^ patients. However, in the first prospective evaluation of MSI in an obstetric cohort, we found that MSI was the same or no better than SI for predicting adverse outcomes related to PPH and sepsis. As MSI is derived from SBP and DBPs, which themselves are calculated from a measured mean arterial pressure from oscillometric BP devices,^26^ it is illogical to assume prediction would improve by this calculation. In addition SBP is more sensitive to haemodynamic shock,^27^ also shown in a systematic review of the relationship between blood loss and clinical signs in obstetric haemorrhage.^28^

Half of the maternal deaths that occur globally are due to bleeding and sepsis^1^ and the vast majority are preventable with relatively affordable, simple interventions, following timely detection. Shock index, a simple concept, has undergone robust prospective evaluation in LMICs where the potential for impact is greatest, and consistently shows superior prediction compared to other vital signs.^12^^,^^16^ SI can be used to ‘rule-in’ and ‘rule-out’ adverse outcomes in the majority of cases, enabling resources to be targeted. Potential clinical utility was demonstrated in a pragmatic cluster randomised control trial in 10 LMIC settings. The CRADLE VSA (incorporating SI) was introduced into maternity care, and a significant reduction in emergency hysterectomies (80%, p = 0.0072) and bleeding-related referrals (O.R 0.56, 95% CI 0.42–0.74) was noted, with no change in deaths from PPH, suggesting that SI facilitated more appropriate referral.^29^ This could, in part, be explained by the fact that SI provides an earlier, and more objective measure of haemodynamic compromise compared to standard PPH definitions based on estimated blood loss, known to be notoriously inaccurate.^30^, ^31^, ^32^ For example, in our study, despite high rates of massive transfusion (≥4 IU), indicating severe haemorrhage, median EBL in the PPH group was 500 mL (IQR 300–800 mL), likely a substantial underestimation.

SI is most likely optimised though integration into care pathways, as recently demonstrated in the ‘EMOTIVE’ trial where the CRADLE VSA (incorporating SI) was in the intervention care bundle which significantly improved PPH identification and led to a lower risk of the primary composite outcome (severe PPH, laparotomy for PPH, maternal death from bleeding) by 60% (RR 0.40; 95% CI, 0.32–0.50; p < 0.0001).^33^ Integration of SI into PPH diagnostic criteria, early-warning scores and care bundles for PPH and sepsis should be prioritised for future work, including implementation studies to explore context specific facilitators and barriers, informing effective adoption, scale-up and policy advancement related to PPH and sepsis management. SI performance was significantly worse in women with co-morbid HDP, and this should be acknowledged if SI is incorporated into clinical pathways. Given the aforementioned global maternal mortality burden associated with HDP, further investigation is needed, including consideration of alternative SI thresholds.

SI has the potential to aid early identification of haemodynamic compromise secondary to PPH and sepsis, enabling targeted allocation of clinical interventions in LMICs, particularly when incorporated into easy-to-use tools, e.g.: CRADLE VSA. This study is timely given the recent global call for a re-appraisal of PPH definitions, and the WHO PPH roadmap which has identified SI as a key research priority to reduce PPH deaths. Shock Index should be evaluated as part of care pathways and algorithms to optimise clinical utility, and could inform ongoing WHO revision of PPH and sepsis definitions and guidelines, enabling earlier PPH and sepsis recognition and improved outcomes across LMIC as well as HIC settings.

## Contributors

All authors contributed to the conception and planning of the work. KK, FJ, RC contributed to carrying out the work. KK led the writing. KK and RC have verified the underlying data. All authors (KK, FJ, RC, AR, CFT, PTS, KB, AHS) contributed to analysis and interpretation of the work, read and approved the final version of the manuscript, and agree to be accountable for all aspects of the work.

## Data sharing statement

De-identified data collected within this study will be made available to researchers after contacting the corresponding author.

## Declaration of interests

We have no competing interests to declare.
