# Supplementary material for: Evaluating shock index for prediction of adverse maternal outcomes related to postpartum haemorrhage and maternal sepsis in Sierra Leone: a prospective observational cohort study
Source: eClinicalMedicine. 2025 Oct 23;89:103578. doi: 10.1016/j.eclinm.2025.103578 (PMC12590223; doi:10.1016/j.eclinm.2025.103578)
Supplement: Supplementary Material [file mmc1.docx]

**Supplementary material**

**Evaluating Shock Index for prediction of adverse maternal outcomes related to postpartum haemorrhage and maternal sepsis in Sierra Leone: A prospective observational cohort study.**

Dr Katy Kuhrt MBBS^a^, Dr Foday Janneh MBBS^b^, Dr Rossetta Cole MBBS^b^, Dr Alexandra Ridout MD^a^, Dr Cristina Fernandez-Turienzo PhD^a^, Mr Paul T. Seed CStat^a^, Dr Kate Bramham FRCP^a^, Professor Andrew H. Shennan FRCOG^a^

^a^ Department of Women and Children's Health, School of Life Course Sciences, Faculty of Life Sciences and Medicine, King's College London, London, UK

^b^ Princess Christian Maternity Hospital, Ministry of Health and Sanitation, Freetown, Sierra Leone

Table S1 First & worst shock index values following diagnosis of postpartum haemorrhage/ sepsis and their timing with respect to diagnosis

| **SI details** | **Postpartum haemorrhage (n=495)** | **Maternal sepsis (n=855)** |
| --- | --- | --- |
| First SI after diagnosis | 1⋅0 ± 0⋅37 | 0⋅97 ± 0⋅30 |
| First HR after diagnosis (bpm) | 104⋅5 ± 21⋅6 | 104⋅4 ± 20⋅5 |
| First SBP after diagnosis (mm/Hg) | 112⋅6 ± 27⋅5 | 113⋅2 ± 24⋅9 |
| Time from diagnosis to first SI (mins) | 0 (-19⋅7 - 0) | 0 (-28⋅1 - 0) |
| *First SI category* | | |
| SI < 0⋅9 | 236, 47⋅7% | 401, 46⋅9% |
| SI 0⋅9 - 1⋅69 | 237, 47⋅9% | 443, 51⋅8% |
| SI ≥1⋅7 | 22, 4⋅4% | 11, 1⋅3% |
| Worst SI after diagnosis | 1⋅20 ± 0⋅35 | 1⋅18 ± 0⋅49 |
| Worst HR after diagnosis | 113⋅03 ± 19⋅3 | 111⋅2 ± 20⋅4 |
| Worst SBP after diagnosis | 98⋅4 ± 20⋅2 | 100⋅1 ± 20⋅4 |
| Time from diagnosis to worst SI (hours) | 6⋅8 (0⋅0 - 27⋅3) | 13⋅4 (0 - 49⋅4) |
| *Worst SI category* | | |
| SI < 0⋅9 | 87, 17⋅6% | 166, 19⋅4% |
| SI 0⋅9 to 1⋅69 | 366, 73⋅9% | 642, 75⋅1% |
| SI ≥1⋅7 | 42, 8⋅5% | 47, 5⋅5% |

*Mean ± standard deviation, median (interquartile range) and n, percentage are shown.*
